# Deciphering the Genetic Architecture of Cooked Rice Texture

**DOI:** 10.3389/fpls.2018.01405

**Published:** 2018-10-02

**Authors:** Gopal Misra, Saurabh Badoni, Cyril John Domingo, Rosa Paula O. Cuevas, Cindy Llorente, Edwige Gaby Nkouaya Mbanjo, Nese Sreenivasulu

**Affiliations:** International Rice Research Institute, Metro Manila, Philippines

**Keywords:** rice texture, multi-locus GWAS, quantitative trait nucleotide, amylose content, adhesiveness, cohesiveness, springiness, hardness

## Abstract

The textural attributes of cooked rice determine palatability and consumer acceptance. Henceforth, understanding the underlying genetic basis is pivotal for the genetic improvement of preferred textural attributes in breeding programs. We characterized diverse set of 236 *Indica* accessions from 37 countries for textural attributes, which includes adhesiveness (ADH), hardness (HRD), springiness (SPR), and cohesiveness (COH) as well as amylose content (AC). A set of 147,692 high quality SNPs resulting from genotyping data of 700K high Density Rice Array (HDRA) derived from the *Indica* diversity panels of 218 lines were retained for marker-trait associations of textural attributes using single-locus (SL) genome wide association studies (GWAS) which resulted in identifying hotspot on chromosome 6 for AC and ADH attributes. Four independent multi-locus approaches (ML-GWAS) including FASTmrEMMA, pLARmEB, mrMLM, and ISIS_EM-BLASSO were implemented to dissect additional loci of major/minor effects influencing the rice texture and to overcome limitations of SL-based GWAS approach. In total 224 significant quantitative trait nucleotide (QTNs) were identified using ML-GWAS, of which 97 were validated with at least two out of the four multi-locus methods. The GWAS results were in accordance with the very significant negative correlation (*r* = −0.83) observed between AC and ADH, and the significant correlation exhibited by AC (*r* < 0.4) with HRD, SPR, and COH. The novel haplotypes and putative candidate genes influencing textural properties beyond AC will be a useful resource for deployment into the marker assisted program to capture consumer preferences influencing rice texture and palatability.

## Introduction

Texture is an important attribute of consumer's acceptance criteria and thus quality evaluation of texture is a critical step in breeding. High range of phenotypic textural variability exist in rice which are described as sticky, soft, dry, firm, and hard. These textural attributes appeals to be of interest to different segment of rice consumers across the globe (Kaosa-Ard and Juliano, 1991). For instance, Japanese and Chinese like the soft or sticky short rice grain, while consumers from Middle East, United States and the Latin America prefer non-sticky firm rice. While the widely preferred target of consumers of India and Pakistan favor soft and fluffy rice varieties (Lyon et al., 2000; Champagne et al., 2010), consumers from Bangladesh, Sri Lanka, Indonesia and Myanmar prefer hard textured rice varieties.

The texture of rice is primarily influenced by the structure and composition of starch in the rice grain. Cooking and eating quality are among the most important components of grain quality routinely assessed in rice breeding programs by three main physicochemical characteristics such as amylose content (AC), gel consistency (GC), and gelatinization temperature (GT) (Ramesh et al., 2000; Zhao and Fitzgerald, 2013; Kong et al., 2015; Cuevas et al., 2016). Primarily, AC is considered as key determinant of rice eating quality (Juliano, 1992). Rice varieties with no or low AC is being linked to sticky and soft texture, respectively (He et al., 1999). Rice cultivars within high AC range showed variable textural attributes (Champagne et al., 1999, 2010). Thus additional parameters such as GC and GT were considered to unravel the degree of hardness (Bhattacharya and Juliano, 1985; Juliano, 1992; Shi et al., 1997; He et al., 1999; Lyon et al., 2000). Though, routine quality parameters found to be useful in predicting cooking quality, these predictive methods do not give sufficient information about the totality of rice textural attributes (Anacleto et al., 2015). Hence, there is a need to explore or develop other rice grain quality metrics which can be used to further differentiate rice texture (Juliano, 1985; Reddy et al., 1994).

Rice texture is regarded as a multidimensional sensory property that perceived by mouth feel characteristic features due to mechanical chewing, rheological, and surface attributes of a product perceptible by means of auditory receptors (Lawless and Heymann, 2010). Mechanical textural attributes of cooked rice such as hardness, cohesiveness, stickiness, and springiness can be characterized by a trained descriptive sensory panel. However, texture assessment pipeline through sensory is not routinely applied during selections of breeding lines because of the low throughput, nature of subjectivity, high cost of training and requirement of maintaining a descriptive panel (Sesmat and Meullenet, 2001). This is particularly true for breeding line selection at the early stages of a rice varietal improvement program, where thousands of lines were subjected for selection. Hence, understanding grain texture has focused on semi-throughput methodologies such as instrumental methods that correlate well with scores reported by sensory panels for the different textural characters (Meullenet et al., 1998; Champagne et al., 1999; Ramesh et al., 2000; Bett-Garber et al., 2001; Mestres et al., 2011). Texture Profile Analysis (TPA) is an instrumental method in which cooked rice grains undergo two compression cycles, mimicking the first and second bites on a food sample and thereby providing information on the mechanical responses such as hardness (HRD), adhesiveness (ADH), springiness (SPR), and cohesiveness (COH) (Stokes et al., 2013). Force-related textural properties of cooked rice can be measured using Texture Analyzer instrument which generate quantitative data, inexpensive, and the results are reproducible and reliable (Ramesh et al., 2000; Mestres et al., 2011).

High diversity in textural properties of rice has been reported (Bao et al., 2006). Like most grain quality traits, phenotypic variation in rice texture is quantitatively inherited (Hori et al., 2016). The genetic complexity of rice texture has been unraveled using classical QTL mapping. Through conventional QTL mapping, TPA parameters have been associated with quantitative trait loci (QTLs) on chromosomes 4 and 5 (HRD), 1 and 7 (ADH), and 8 (SPR) in a recombinant inbred mapping population whose individuals have low AC (Cho et al., 2010). So far these reported QTLs influencing texture have not been fine mapped and candidate genes not identified yet. These findings are notable because AC, coded by the *Waxy* gene in chromosome 6, is known to correlate positively with HRD and negatively with ADH (Suwannaporn et al., 2007); yet, no significant associations were reported by Cho et al. (2010), indicating that other genes are contributing to these textural attributes in cooked rice. This warrants the need of precise and robust statistical approaches for efficient capturing major with minor effect loci influencing the rice texture, which would pave the way to better understand the underlying genetic architecture.

More recently, genome wide association studies (GWAS) has become the state-of-art method to link genotypic variation to corresponding differences in phenotype, with the aim of dissecting the genetic basis of complex trait in various crops (Ingvarsson and Street, 2011; Xiao et al., 2017). GWAS offers high resolution-mapping by utilizing the historical recombination events, which leverages it with identification of key allelic variants and haplotypes in the underlying candidate genes. Nevertheless, single locus approach fails to consider the integrated effect of multiple markers under specific loci (Wang et al., 2016; Tamba et al., 2017). Moreover, using too conservative Bonferroni correction minimizes the likelihood to detect many important small effect loci (Wen et al., 2018). These issues have been addressed by efficiently utilizing the multi-locus GWAS approach in recent studies (Segura et al., 2012; Liu et al., 2016; Wang et al., 2016; Tamba et al., 2017; Wen et al., 2018; Zhang et al., 2018).

In addition to single locus genome wide association, the multi-locus GWAS methods were performed in the present study to overcome the limitations of single locus–based GWAS and to define the genetic basis of cooked rice texture and grain amylose content traits in *Indica* diversity lines. Furthermore, we conducted targeted gene-based association study using the available SNPs in the neighborhood region, which led to the construction of haplotypes showing phenotypic variation for the texture component traits.

## Materials and methods

### Plant materials

A total of 236 diverse *Indica* accessions were selected from the rice diversity panels (RDP) (McCouch et al., 2016) by ensuring that the days to maturity is close between all entries, which did not exceed 140 days. These germplasm lines have been grown at the Robert S. Zeigler Experiment Station (ZES) of the International Rice Research Institute (IRRI), Laguna, Philippines (14°N, 121°E) during the 2014 dry season and wet season in randomized block design in three replications. Standard uniform field and crop management procedures have been adopted based on IRRI standard procedure across all of the replicates. Harvesting was done in the month of May/June depending upon their maturity time. After harvesting, standard IRRI drying method was followed in order to attain 12–14% seed moisture content. Subsequently, seeds were stored in the brown double-layer seed paper bags inside the seed storage room maintained at 18°C with optimum relative humidity.

### Sample processing

Paddy rice samples were dehulled using THU-35A test dehusker (Satake Corp., Japan) and brown rice was milled through Grainman 60-230-60-2AT instrument, (Grain Machinery Mfg. Corp., USA) to produce white milled rice samples. A portion from each sample corresponding to 100 unbroken grains was used for texture profile analyses (TPA) and the rest of samples were ground to a fine powder using Cyclone Sample Mill 3010-030, Udy Corp, USA. The resulted homogenized rice flour was further used for estimating amylose content (AC).

### Amylose content measurement

AC determination was based on iodine colorimetric reaction using method of ISO 6647 (International Organization for Standardization, 2007) on milled rice flour. Briefly, gelatinized flour suspension was injected into the glass transition lines of a San++ Segmented Flow Analyser (SFA) system (Skalar Analytical B.V., The Netherlands) and allowed to react with iodine to form amylose-iodine complex (K-I_2_). The absorbance of the sample's containing K-I_2_ complex was estimated at 620 nm wavelength and subsequently, AC was quantified with standards by plotted against the standard curve.

### Texture profile analysis (TPA)

Twenty-five whole polished rice grains per sample of accession were washed thrice, soaked for 30 min in Milli-Q water (1 mL) for 15 min in a test tube. The samples were heated to boiling point for 20 min and kept at 50°C prior to avoid retrogradation. Textural parameters of the cooked rice (hardness, cohesiveness, springiness, and adhesiveness) were analyzed according to the method described by Lyon et al. (2000) with modifications. The Ta.XT-Plus Texture Analyzer (Stable Micro Systems Ltd., Surrey, UK), equipped with a 35-mm aluminum cylinder probe with a 5-kg load cell, was used. The probe was positioned 15 mm above the base. Three intact cooked rice kernels were placed parallel with each other on the aluminum plate base under the center of the probe and compressed to 90% of their original height. The TPA force-deformation curve was obtained using a two-cycle compression test. The instrument is set with a test and post-test speed of 0.5 mm s^−1^. Values of HRD (peak force of the first compression by the height of first curve), ADH (Negative force area under the first bite), COH (A_2_/A_1_), and SPR (T_2_/T_1_) were obtained and processed using Exponent Lite Software (version 3.0.5.0). ADH was recorded as negative numbers to indicate the direction of the probe's movement. Hence, adhesiveness values were reported in absolute values. Texture experiments were conducted in triplicate with three biological replications. For further details, refer Supplementary Note 1.

### Genotyping dataset

A 700K high Density Rice Array (HDRA) SNP genotyping set developed by an Affymetrix Custom Gene Chip Array from a SNP discovery dataset (McCouch et al., 2016) was used to develop genotyping information from the panel of 236 cultivars. A total of distinct 218 diverse germplasm lines were selected from the panel of 236 cultivars after following the standard filtering criterion with a missing rate of not more than 10% (mind 10%, geno 10%) and a minor allele frequency of at least 5%. This resulted into the consideration of final set of 147,692 high quality SNPs for conducting GWAS.

### Single-locus and multi-locus genome wide association studies (GWAS)

Mixed linear model based EMMAx (Kang et al., 2010) was carried out to conduct single locus (SL)-GWAS pipeline (Butardo et al., 2017). WarpedLMM (Fusi et al., 2014) was used to transform the phenotype to fulfill the normally distributed phenotype data for conducting the mixed linear model based approach. EMMAx-kin function was used to create the kinship matrix. Furthermore, Manhattan plot and Q-Q plot were created using the R package qqman (Turner, 2018). The Bonferroni corrected *p*-value [−log_10_(*P*) = 6.47; *P* = 0.05/147692] was used as a threshold *p*-value. Nevertheless, since few loci have surpassed this threshold, significant SNPs above suggestive line at *p*-value of utmost 1e-5 were extracted as set of significant SNPs identified from SL-GWAS approach. Linkage Disequilibrium (LD)-plot and beta-effects of SNPs were plotted using combination of Haploview (Barrett et al., 2005) and Rscript. Targeted associations were done for the selected genes based on LD block and defined significant level. Annotations of candidate genes are based on MSUv7 annotation. Genetic regions identified from SL-GWAS approach were validated using at least two independent methods of ML-GWAS.

Four different methods namely FASTmrEMMA (Wen et al., 2018), pLARmEB (Zhang et al., 2017), mrMLM (Wang et al., 2016), and ISIS_EM-BLASSO (Tamba et al., 2017) were used to conduct multi-locus GWAS on 218 diverse germplasm with 147,692 high quality SNPs. All parameters used at their default values of respective method (Misra et al., 2017). A SearchRadius parameter (bin) (https://cran.r-project.org/web/packages/mrMLM/mrMLM.pdf) size of 20 SNPs was used as parameter to run the multi-locus association using algorithms mrMLM and FASTmrMLM. A threshold criterion of LOD of 3 and above was used to get the final set of significant SNPs. In house perl script was used to identify the overlapping genes with significant LOD score SNPs. SNPEff (Cingolani et al., 2012) was used for identifying the functional annotation of the respective SNP. Through implication of multi-locus GWAS and targeted associations, the boxplots were created to visualize the phenotype distribution among constructed haplotypes. Using R program, boxplots depicting phenotype distributing ranges were plotted for the respective textural traits. Multiple-*t*-test (pair-wise comparison) was implemented to identify the boxplot with significant phenotypic value at the significant level of *P* ≤ 0.05.

## Results

### Correlations between AC and texture affecting traits

Texture profile of 236 *Indica* diversity lines was evaluated. However, owing to follow high quality genotypic information upon filtering, a total of 218 diversity lines originated from 35 countries were selected to study AC and texture associated traits encompassing ADH, COH, HRD, and SPR. The pattern of phenotypic values for AC and ADH was skewed as many *Indica* germplasm found to be enriched for intermediate and high AC. Conversely, broad range of phenotypic values was observed for HRD, COH, and SPR attributes (Figure S1). Cor function with Pearson's method was used to detect the correlation and corrplot was used to create the plot.

**Figure 1:**
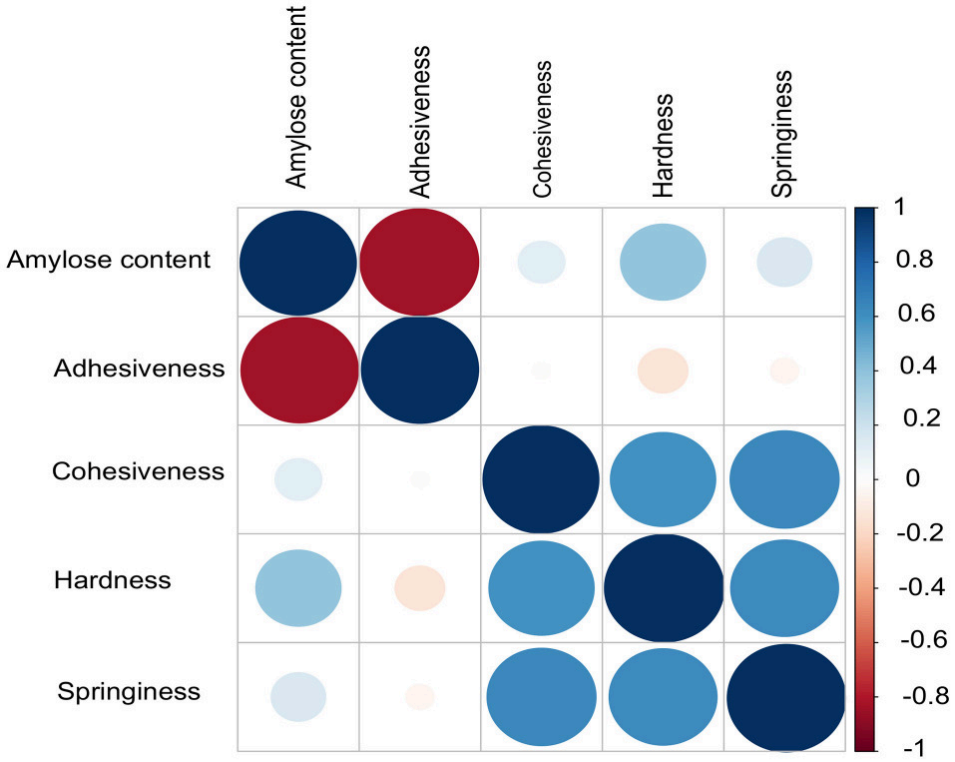
Correlations among different grain quality attributes contributing to texture including the amylose content. Pearson method was used to calculate the correlation. Adhesiveness (ADH) and amylose content (AC) inversely correlated. No correlation was observed between AC other textural parameters cohesiveness (COH), hardness (HRD) and springiness (SPR).

AC exhibited a high negative correlation with ADH (*r* = −0.83, *P* = 2.2E^−16^), which supports the fact that low amylose rice accessions tends to be stickier (Figure 1). Although, AC was considered as a key selection criterion for predicting cooked rice texture in normal breeding practices, HRD showed weak positive correlation with AC (*r* = 0.39, *P* = 3.05E^−09^). Moreover, we did not observe significant correlation between AC with other textural attributes viz. COH and SPR. Instead, we observed a positive correlation within 3 textural attributes such as COH, HRD, and SPR (*r* > 0.59, *P* = 2.2E^−16^; Figure 1).

### Genetic dissection of texture related traits using single-locus genome wide association studies (SL-GWAS)

**Figure 2:**
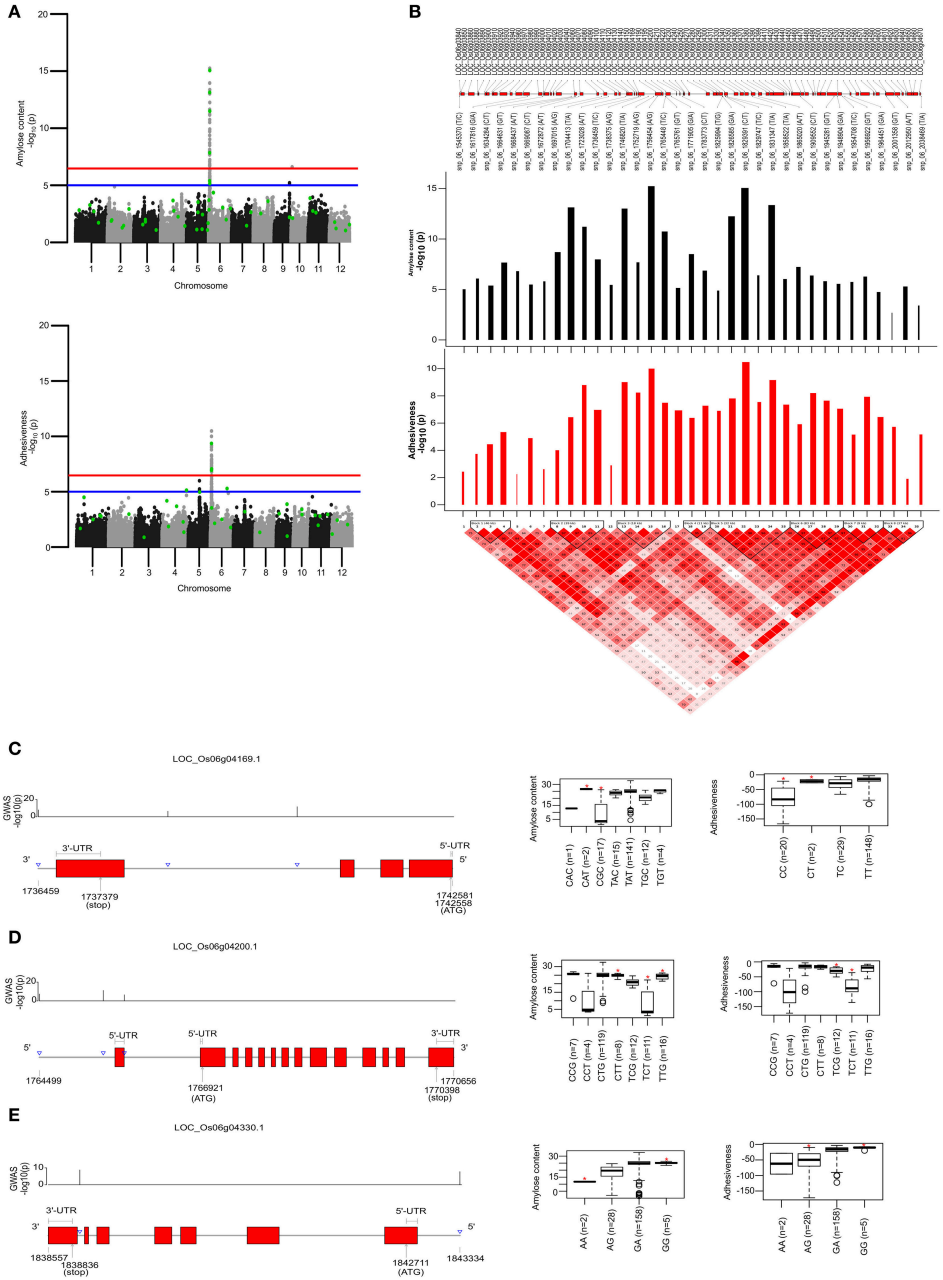
Single-locus (SL) and multi-locus (ML)-GWAS for AC and ADH. **(A)** Manhattan plot showing QTNs identified from SL-GWAS showed in gray/black and ML-GWAS QTNs highlighted in green dot within the Manhattan plot. Genome-wide significant threshold line [–log_10_(*P*) = 6.47] is drawn as red, whereas suggestive line is represented by blue line at –log_10_(*P*) of 5. **(B)** Linkage Disequilibrium (LD) plot with tagged SNPs from the pool of significant SNPs over suggestive line were plotted. A total of 8 blocks were identified based on D′ threshold criterion equal to 0.8. The –log_10_(*P*) values was plotted as bar plot with positive effect as black bars and negative effect with red bars where width of bars represent the phenotypic effect size termed as beta effect. The overlapping genes were plotted in the top most lane **(C–E)**. Targeted gene associations for LOC_Os06g04169, LOC_Os06g04200, and LOC_Os06g04330 present in second, third and fifth LD-block, respectively. Gene structure with significant SNPs and phenotype distribution as boxplot were presented. An asterisks (*) represented the haplotype with significant phenotypic value (at significance level of *P* ≤ 0.05) using pair wise *t*-test.

**Figure 3:**
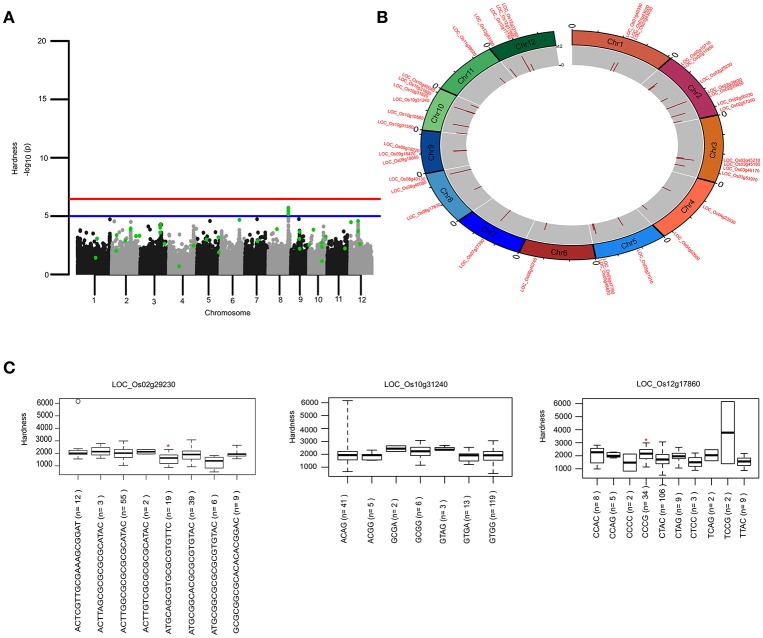
Genetic regions identified through ML-GWAS and SL-GWAS for hardness (HRD). **(A)** Manhattan plot showing the multi-locus associations (QTNs with LOD ≥3; highlighted as green dots) overlaid with SL-GWAS QTNs (black/gray) for HRD. **(B)** Circos representing the physical positioning of 12 chromosomes with locus IDs of significant QTNs identified in ML-GWAS, followed by depiction of LOD score in the innermost circle. **(C)** Phenotypic distribution of haplotypes shown as boxplot for selected genes identified from ML-GWAS method. Haplotypes showing significant HRD values were highlighted with an asterisks (*) (at the significance level of *P* ≤ 0.05) using pair wise *t*-test.

**Figure 4:**
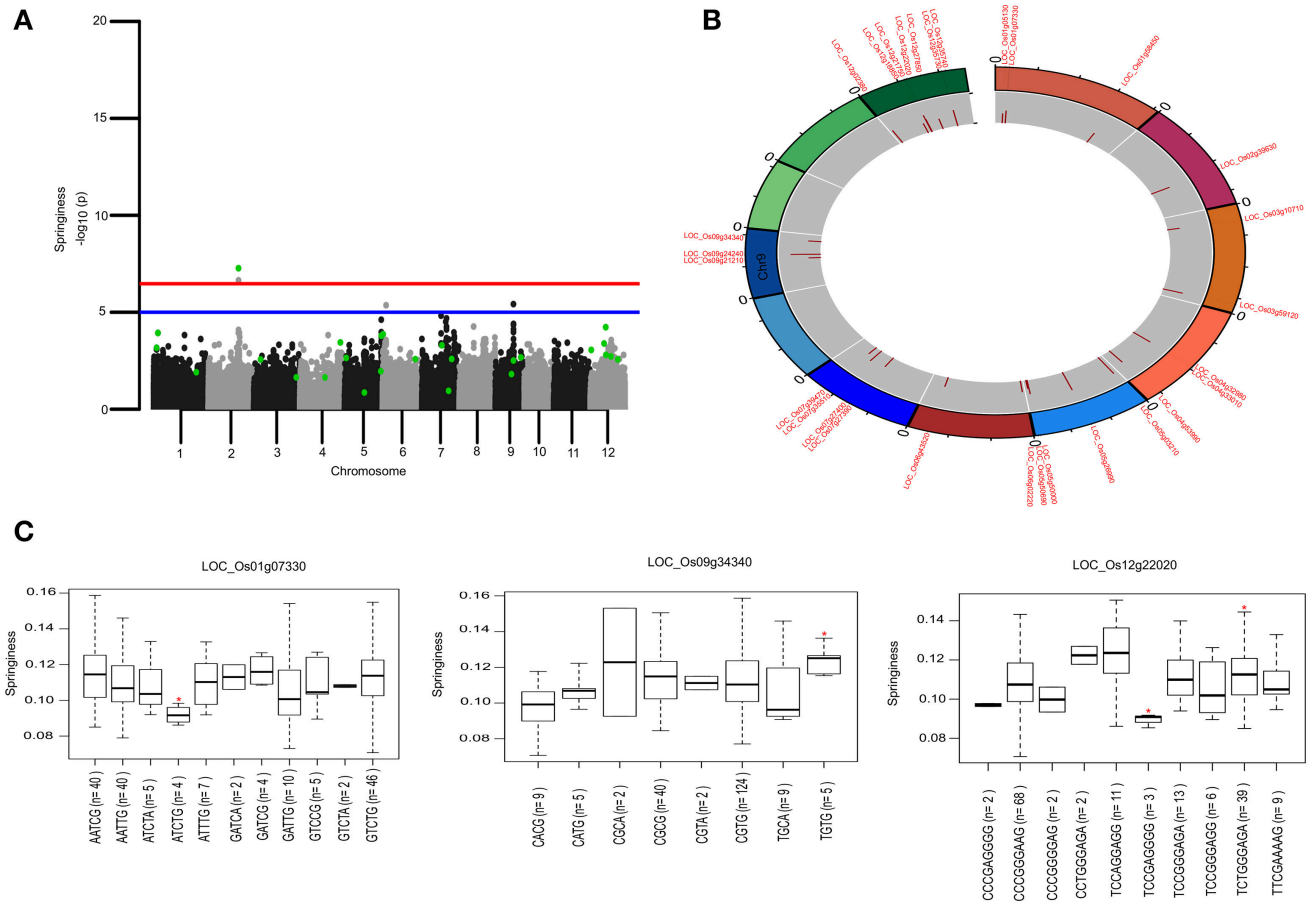
Genetic regions identified through ML-GWAS and SL-GWAS for springiness (SPR). **(A)** Manhattan plot representing the multi-locus associations (QTNs with LOD ≥3; highlighted as green dots) overlaid with SL-GWAS QTNs (black/gray) for SPR. **(B)** Circos representing the physical positioning of 12 chromosomes with locus IDs of significant QTNs identified in ML-GWAS, followed by depiction of LOD score in the innermost circle. **(C)** Phenotypic distribution of haplotypes shown as boxplot for selected genes identified from ML-GWAS method. Haplotypes showing significant SPR values were highlighted with an asterisks (*) (at the significance level of *P* ≤ 0.05) using pair wise *t*-test.

**Figure 5:**
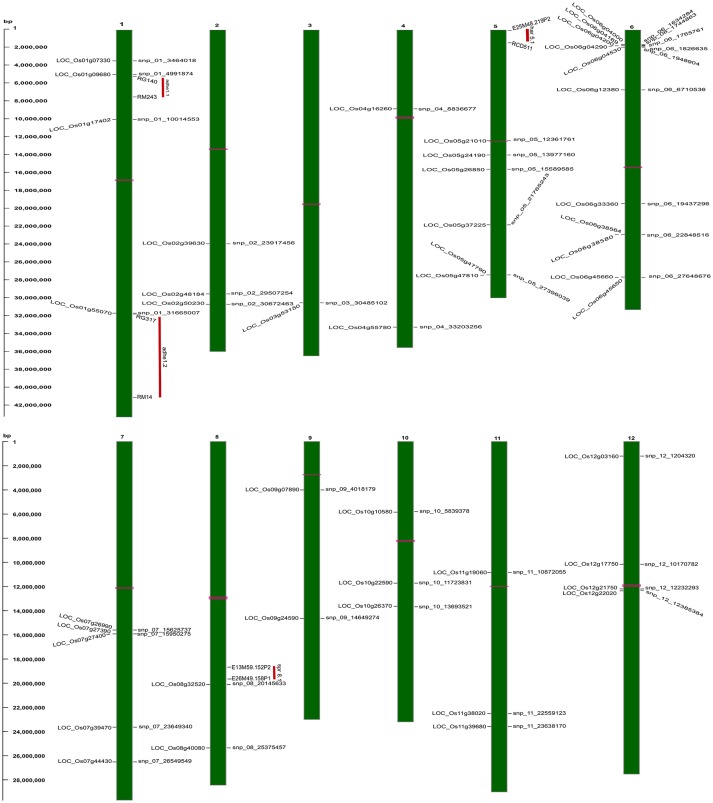
Physical map of strongly associated QTNs on rice chromosomal maps identified using ML-GWAS methods. The represented QTNs on map includes either QTNs jointly identified using SL- and ML-GWAS, or spotted in at least two independent ML-GWAS methods, mapped at their physical positions on 12 chromosomes. On the left, scale indicates the base pair (bp) distance. Publicly available texture QTL information from Cho et al. (2010) have been mapped, and were highlighted as vertical red colored bars aligning with respective physical positions on the map. Horizontal bars (in pink color) on chromosomal maps represent the position of centromere.

To identify the large effect genetic regions of textural attributes in cooked rice and to delineate its interrelationship with AC, single-locus (SL) GWAS was performed using EMMAx (Kang et al., 2010) for marker trait associations in 218 germplasm lines using high quality 147,692 SNPs from the high density rice array (HDRA) panel of 700 K SNPs (McCouch et al., 2016). A total of 131 loci were associated [above suggestive lines; −log_10_(*P*) ≥ 5] with texture-related traits. Among them the prominent peak on chromosome 6 associated with highly heritable traits AC (*h*^2^ = 0.86) and ADH (*h*^2^ = 0.86) (Figure 2), as indicated with the threshold value of significance by a red horizontal line in the Manhattan plot at –log_10_(*P*-value) = 6.39. HRD is another highly heritable trait (*h*^2^ = 0.82), a significant region [–log_10_(*P*) > 5; blue suggestive line] was detected on chromosome 8 (Figure 3). Genetic association of SPR found for loci on chromosome 2, 6, and 9 were statistically significant (Figure 4). No QTN significantly associated with cohesiveness was detected utilizing SL-GWAS (Figure S2). Therefore, we furthermore employed multi-locus GWAS to reveal the significant loci including the small-effect loci (Figure 5).

### Genetic dissection of textural attributes employing multi-locus -genome wide association studies (ML-GWAS)

For identifying novel associations and validation of loci detected using SL-GWAS, the multi-locus (ML) model approach was followed utilizing four independent methods namely FASTmrEMMA (Wen et al., 2018), pLARmEB (Zhang et al., 2017), mrMLM (Wang et al., 2016), and ISIS_EM-BLASSO (Tamba et al., 2017). The multi-locus method pLARmEB identified the highest number (90) of associated SNPs, followed by ISIS-EM-BLASSO (57). The lowest number of SNPs linked to texture related-traits was obtained with the FASTmrEMMA method (Tables S1, S2). A total of 173 quantitative trait nucleotides (QTNs) associated with texture attributes including AC, ADH, COH, HRD, and SPR were identified on all 12 chromosomes (Tables S1, S2). Notably, highly associated 10 QTNs identified with SL-GWAS method were validated using ML-GWAS methods (Table 1). These candidate genes includes QTNs on chromosome 2 regulating SPR (LOC_Os02g39630) and chromosome 4 QTN (LOC_Os04g55780) associated with ADH. An 87kb fine-mapped region (25.38–25.46 Mb) on chromosome eight identified QTN (LOC_Os08g40080) linked with HRD. Interestingly, hot spot QTLs on chromosome 6 co-located for two traits linking ADH textural trait (LOC_Os06g04169, LOC_Os06g04200, LOC_Os06g04530, intergenic region covering LOC_Os06g38564-LOC_Os06g38580) with AC (LOC_Os06g04000, LOC_Os06g04200, LOC_Os06g04290) validated using SL-GWAS and ML-GWAS methods (Table 1).

**Table 1:** Significant SNPs associated with amylose content and texture-related traits identified using single and multi-locus GWAS methods.

**SNP_ID**	**Chr**	**Method**	**LOD**	***r*^2^ (%)**	**Trait**	**Beta**	**–log_10_(p)**	**Gene**	**Nucleotide change**	**SNP location/Amino acid change**	**SWISSPROT**
snp_02_23917456	2	mrMLM	5.68	10.27	SPR	0.45	7.27	LOC_Os02g39630	c.*4252G>A	Downstream gene	No hits
snp_04_33203256	4	mrMLM, pLARmEB	3.21; 6.38	0.84; 0.16	ADH	−0.42	5.13	LOC_Os04g55780	c.-3159G>A	Promoter region	Arogenate dehydratase/prephenate dehydratase
snp_06_1634284	6	ISIS-EM-BLASSO mrMLM, pLARmEB	3.08; 12.48; 22.33	4.11; 9.03; 1.50	AC	0.59	5.39	LOC_Os06g04000	c.-895C>T	Promoter region	Peptidyl-prolyl cis-trans isomerase
snp_06_1744863	6	mrMLM, pLARmEB	7.88; 29.90	3.25; 1.37	ADH	−0.61	9.36	LOC_Os06g04169	c.-2305C>T	Promoter region	No hits
snp_06_1765761	6	FASTmrEMMA pLARmEB	11.69; 12.18	5.64; 1.11	AC	0.54	5.15	LOC_Os06g04200	c.-33+2G>T	Splice variant	Granule-bound starch synthase1
snp_06_1765761	6	FASTmrEMMA	12.32	7.77	ADH	−0.63	6.94	LOC_Os06g04200	c.-33+2G>T	Splice variant	Granule-bound starch synthase1
snp_06_1826635	6	ISIS-EM-BLASSO pLARmEB	12.36; 23.59	3.69; 0.84	AC	0.64	11.49	LOC_Os06g04290	c.-4704G>T	Promoter region	40S ribosomal protein S20
snp_06_1948904	6	mrMLM, FASTmrEMMA, ISIS_EM-BLASSO	5.57; 14.91; 16.38	1.87; 8.05; 5.86	ADH	−0.53	7.06	LOC_Os06g04530	c.-601G>A	Promoter region	ATP-dependent Clp protease
snp_06_22848516	6	pLARmEB, ISIS_EM-BLASSO, mrMLM	3.33; 3.37; 5.55	0.08; 1.26; 1.69	ADH	−0.56	5.29	LOC_Os06g38564-LOC_Os06g38580	n.22848516G>C	Intergenic region	No hits
snp_08_25375457	8	pLARmEB, mrMLM	4.27; 6.51	1.68; 4.56	HRD	0.36	5.17	LOC_Os08g40080	c.-181C>T	Promoter region	No hits

In addition, we observed a number of unique 38 QTNs associated with texture identified by two or more independent methods of ML-GWAS (Table 2) indicated with green dots in the Manhattan plot, which could not be otherwise detected by SL-GWAS due to adoption of higher threshold criterion (Figure 2). ML-GWAS enabled detection of 3 QTNs regulating both AC and ADH on chromosome 1 (LOC_Os01g09680), chromosome 4 (LOC_Os04g55780), and major effect genetic loci LOC_Os06g04200 from chromosome six encompassing the *GBSS1* (Waxy) gene (Table 2, Figures 2A,B). Additionally, a highly significant SNP found on chromosome 12 (LOC_Os12g22020, intronic splice variant) being associated with COH (LOD score of 6.35 with *r*^2^ value of 12.05) and SPR (LOD score of 4.96 with *r*^2^ value of 2.89). Additional SNPs associated with ADH was identified on chromosome 1 (LOC_Os01g17402) with a highly significant SNP of splice variant in Cyclin B1-3 and on chromosome 5 (LOC_Os05g26850, promoter region of unknown gene), identified by all four multi-locus methods (Table 2).

**Table 2:** Significant SNPs associated with amylose content and texture-related traits identified using several multi-locus GWAS method.

**SNP_ID**	**Chr**	**Method(s) used**	**LOD**	***r*^2^ (%)**	**Trait**	**Gene**	**Nucleotide change**	**SNP location/Amino acid change**	**SWISSPROT**
snp_01_3464018	1	pLARmEB; FASTmrEMMA	3.38; 4.63	0.76; 4.04	SPR	LOC_Os01g07330	c.-187A>G	Promoter region	No hits
snp_01_10014553	1	pLARmEB; ISIS_EM-BLASSO; FASTmrEMMA; mrMLM	4.50; 5.36; 7.97; 13.09	0.33; 3.96; 5.55; 6.54	ADH	LOC_Os01g17402	c.-3673C>A	p.Val140Ile	No hits
snp_01_31665007	1	FASTmrEMMA; mrMLM; pLARmEB	3.76; 8.18; 17.48	1.65; 3.04; 0.74	ADH	LOC_Os01g55070	c.-3673C>A	Downstream gene	No hits
snp_01_4991874	1	ISIS_EM-BLASSO; FASTmrEMMA	3.51; 10.29	0.76; 2.43	AC	LOC_Os01g09680	c.212-4G>A	Promoter region	Cyclin-B1-3
snp_01_4991874	1	FASTmrEMMA	5.98	2.53	ADH	LOC_Os01g09680	c.-926C>T	Promoter region	Pentatricopeptide repeat containing protein
snp_02_23917456	2	pLARmEB	10.44	3.08	HRD	LOC_Os02g39630	c.*4252G>A	Intergenic region	No hits
snp_02_29507254	2	pLARmEB; mrMLM	3.82; 6.21	0.08; 2.26	AC	LOC_Os02g48184	c.-5090C>T	Promoter region	No hits
snp_02_30672463	2	mrMLM; pLARmEB	6.89; 6.90	7.41; 2.15	HRD	LOC_Os02g50230	c.1227C>T	p.Ala399Val	No hits
snp_03_30485102	3	pLARmEB; FASTmrEMMA	3.60; 4.42	0.08; 1.36	AC	LOC_Os03g53150	c.-1973G>A		Auxin-responsive protein_IAA13
snp_04_33203256	4	pLARmEB; ISIS_EM-BLASSO	5.82; 8.86	0.13; 2.15	AC	LOC_Os04g55780	c.-2831T>C	Intergenic region	No hits
snp_04_8836677	4	mrMLM; pLARmEB	4.57; 13.14	1.38; 0.48	ADH	LOC_Os04g16260	c.-3159G>A	Promoter region	Arogenate dehydratase/prephenate dehydratase
snp_05_12361761	5	pLARmEB; ISIS_EM-BLASSO	4.36; 5.47	3.07; 10.70	HRD	LOC_Os05g21010	c.418G>A	p.Val140Ile	No hits
snp_05_13977160	5	ISIS_EM-BLASSO; pLARmEB	3.42; 6.05	1.26; 0.27	AC	LOC_Os05g24190	c.*3646T>C	Downstream gene	polyprotein
snp_05_15589585	5	ISIS_EM-BLASSO; FASTmrEMMA; mrMLM; pLARmEB	6.11; 11.45; 13.72; 26.36	3.05; 5.03; 4.72; 1.22	ADH	LOC_Os05g26850	c.-2404T>A	Promoter region	No hits
snp_05_21765243	5	pLARmEB; FASTmrEMMA	4.66; 13.75	0.11; 4.42	AC	LOC_Os05g37225	c.-2713C>T	Promoter region	No hits
snp_05_27396039	5	mrMLM; ISIS_EM-BLASSO	3.82; 6.36	2.29; 3.77	HRD	LOC_Os05g47790-LOC_Os05g47810	n.27396039A>G	Intergenic region	No hits
snp_06_19437296	6	mrMLM; ISIS_EM-BLASSO; pLARmEB	3.92; 4.15; 6.69	1.47; 1.15; 0.17	AC	LOC_Os06g33360	c.-1942C>T	Promoter region	No hits
snp_06_27648676	6	pLARmEB; mrMLM	6.72; 7.82	0.32; 3.00	ADH	LOC_Os06g45650-LOC_Os06g45660	c.1196C>T	p.Ala399Val	NB-ARC_domain-containing protein
snp_06_6710536	6	pLARmEB; mrMLM	4.56; 7.11	0.12; 2.00	AC	LOC_Os06g12380	n.27648676A>G	Intergenic region	No hits
snp_07_15625737	7	mrMLM; pLARmEB	3.38; 6.96	1.29; 0.21	ADH	LOC_Os07g26990	c.52G>A	p.Thr18Thr	No hits
snp_07_15950275	7	pLARmEB	4.01	0.7	SPR	LOC_Os07g27390-LOC_Os07g27400	n.15950275C>T	Intergenic region	No hits
snp_07_15950275	7	ISIS_EM-BLASSO; pLARmEB	4.61; 7.83	3.56; 2.23	HRD	LOC_Os07g27390-LOC_Os07g27400	n.15950275C>T	Intergenic region	No hits
snp_07_23649340	7	ISIS_EM-BLASSO	3.06	1.5	SPR	LOC_Os07g39470	c.-2786T>G	Promoter region	Chitin-inducible gibberellin responsive gene
snp_07_26549549	7	pLARmEB	5.59	0.06	AC	LOC_Os07g44430	c.513C>T	p.Asp171Asp	phospholipase
snp_08_20145633	8	FASTmrEMMA; pLARmEB	5.90; 6.40	1.03; 0.09	AC	LOC_Os08g32520	c.*441G>A	Downstream gene	No hits
snp_09_14649274	9	pLARmEB; ISIS_EM-BLASSO; mrMLM	3.72; 3.80; 4.7	0.15; 1.82; 2.42	ADH	LOC_Os09g24590	c.-3712C>T	Promoter region	polyprotein
snp_09_4018179	9	ISIS_EM-BLASSO; pLARmEB; mrMLM	3.60; 4.18; 6.67	1.32; 0.11; 1.91	ADH	LOC_Os09g07890	c.*523A>T	3 prime UTR	No hits
snp_10_11723831	10	FASTmrEMMA; mrMLM	4.07; 6.25	1.27; 1.43	ADH	LOC_Os10g22590	c.-2300C>T	Promoter region	No hits
snp_10_13693521	10	ISIS_EM-BLASSO; FASTmrEMMA	3.69; 5.76	10.69; 0.00	COH	LOC_Os10g26370	c.187+112C>A	Intron	allergen and extensin family protein
snp_10_5839378	10	mrMLM; pLARmEB	4.12; 7.89	8.67; 5.85	HRD	LOC_Os10g10580	c.*4639A>T	Downstream gene	No hits
snp_11_10872055	11	pLARmEB; ISIS_EM-BLASSO	4.29; 5.27	0.16; 2.56	AC	LOC_Os11g19060	c.*3782C>T	Downstream gene	AP2-like ethylene-responsive transcription famylose contenttor
snp_11_22559123	11	ISIS_EM-BLASSO; pLARmEB	3.35; 3.93	1.74; 0.80	HRD	LOC_Os11g38020	c.1110+4C>T	Splice variant	small GTP-binding protein
snp_11_23638170	11	ISIS_EM-BLASSO; FASTmrEMMA	3.71; 4.50	1.10; 1.49	ADH	LOC_Os11g39680	c.*2157G>A	Downstream gene	No hits
snp_12_10170782	12	pLARmEB; ISIS_EM-BLASSO; mrMLM	6.29; 8.71; 10.25	2.26; 7.76; 10.68	HRD	LOC_Os12g17750	c.830G>A	p.Gly277Asp	No hits
snp_12_1204320	12	FASTmrEMMA; mrMLM	3.10; 5.90	3.24; 5.81	HRD	LOC_Os12g03160	c.-4154C>T	Promoter region	No hits
snp_12_12232293	12	mrMLM; pLARmEB	3.62; 6.64	11.00; 0.27	SPR	LOC_Os12g21750	c.-4282G>C	Promoter region	No hits
snp_12_12385384	12	ISIS_EM-BLASSO	4.96	2.89	SPR	LOC_Os12g22020	c.175-375G>A	Intron	No hits
snp_12_12385384	12	ISIS_EM-BLASSO	6.35	12.05	COH	LOC_Os12g22020	c.175-375G>A	Intron	No hits

### Major genomic region determines the adhesiveness (ADH) and amylose content (AC)

AC being the starch component, it is one of the key determinants of the cooking and eating quality. In cooked form, AC negatively influences the ADH (Figure 1). Major genetic region of ~490 kb candidate genomic region (1.54–2.0 Mb) possessing 8 LD-blocks on chromosome 6 has been mapped for both ADH and AC, confirmed using both SL-GWAS and ML-GWAS methods (Table 1, Figures 2A,B, Figure 3). Interestingly, this fine mapped genetic region on chromosome 6 was consistently identified when GWAS has been conducted across wet and dry seasons (Figure S3). Notably, multi-loci GWAS detected moderate to high effect significant SNPs from LD blocks 2, 3, and 5 defining the variations for AC and ADH (Figure 2B). Furthermore, haplotypes identified from LD-block 2 and 3 contributed in distinguishing samples from high/intermediate amylose classes with low AC (Figures 2C,D), whereas allelic variants from LD-block 5 differentiates lines possessing intermediate AC with low AC (Figure 2E). Most haplotypes showing the high AC (>25%) were observed as less adhesive (low magnitude) as reflected in the haplotypes from LD-block 2 and 3. Likewise, haplotypes fixed for intermediate AC, showed moderate variations for ADH. Contrastingly, low AC samples showed high ADH values, as observed in LD-block 5. Targeted-gene association study of the potential candidate genes (LOC_Os06g04169 encoding beta-hydrolase, LOC_Os06g04200 identified as *GBSS1* and LOC_Os06g04330 annotated as Phosphotransferase) distinguished different AC and ADH classes of phenotypes (Figures 2C,E, Table S2), were validated by two independent methods such as ML-GWAS and SL-GWAS. For the candidate gene LOC_Os06g04169, haplotype CGC were found to be correlated to low AC and high ADH phenotypes and its alternative haplotype (TAC/TAT/TGC/TGT) containing lines were correlated to possess high AC and low ADH (Figure 2C). Haplotype (CCT/TCT) identified from the LOC_Os06g04200 were correlated to low AC and high ADH phenotypes and its alternative haplotypes (CTG/CTT/TTG) containing lines found to possess high AC and low ADH (Figure 2D).Within LOC_Os06g04200 (granule bound starch synthase I), the high effect QTN at position 1765761 (LOD > 11) lying at splice junction of exon 1 detected using FASTmrEMMA and pLARmEB significantly affected both AC and ADH values (Table S2). Additionally, splice junction QTNs along with other two QTNs identified in *GBSS1* showed variable level of allele frequencies (Table S3). Conversely, haplotypes from candidate gene LOC_Os06g04330 explained the variations among intermediate and low AC classes (Figure 2E).

Utilizing the SL and ML-GWAS, prominent association signals were identified for ADH alone from chromosome 4 (LOC_Os04g55780), which is linked negatively with ADH trait with a beta value of −0.42 identified using SL-GWAS and confirmed using two independent methods of ML-GWAS with LOD scores of 3.2 and 6.4. In addition, a total 11 QTNs were identified for ADH but not linked to AC, which are identified from at least two independent methods of ML-GWAS (Figure 5). These candidate loci of major effect QTNs affecting ADH trait located on chromosome 1 (LOC_Os01g09680 and LOC_Os01g55070 with *r*^2^ value of 2.53 and 1.65, respectively), chromosome 4 (LOC_Os04g16260 with r^2^ value of 1.38), chromosome 5 (LOC_Os05g26850 with *r*^2^ value of 3.05), chromosome 7 (LOC_Os07g26990 with *r*^2^ value of 1.29), chromosome 9 (LOC_Os09g07890 with *r*^2^ value of 1.32), chromosome 10 (LOC_Os10g22590 with *r*^2^ value of 1.27), and chromosome 11 (LOC_Os11g39680 with *r*^2^ value of 1.10) (Table 2). Using four different methods, multi-locus GWAS yielded highly significant (LOD > 10) SNP with splice variant in candidate gene LOC_Os01g17402 and in the promoter region of LOC_Os05g26850, explaining high heritable phenotypic variation for traits AC and ADH (Table S2). Among them highly significant snp_05_15589585 (c.-2404T>A) present in the upstream of LOC_Os05g26850 (unclassified) on chromosome 5 showed prominent association with ADH using different ML-GWAS methods, while it was not significant under SL-GWAS (Table S2).

Additional 11 QTNs were identified to influence AC but not linked to ADH confirmed from two or more independent methods of ML-GWAS. With the exception of 3 QTNs (LOC_Os05g24190, LOC_Os06g33360, and LOC_Os08g32520), many of them were turned out to be minor effect QTNs with low r^2^ value. The major effect QTN affecting AC namely snp_06_19437296 has a non-synonymous nucleotide change (C>T) in the candidate gene LOC_Os06g33360 lead to amino acid change (Ala>val) (Table 2).

### Genetic dissection of hardness (HRD), cohesiveness (COH), and springiness (SPR) as components of textural attributes

The key components determining the cooked rice texture include the HRD, COH, and SPR, which showed higher correlations among each other (Figure 1), but not linked to AC variation in the grain. Significant QTN on chromosome 8 with SNP (C181>T) found in the promoter region of LOC_Os08g40080 influence HRD, validated by both SL-GWAS and ML-GWAS methods (Table 1). For HRD, multi-locus GWAS yielded 9 significant QTNs identified on chromosomes 2 (LOC_Os02g39630, LOC_Os02g50230), chromosome 5 (LOC_Os05g21010, intergenic region interval LOC_Os05g47790-LOC_Os05g47810), chromosome 7 (intergenic interval LOC_Os07g27390-LOC_Os07g27400), chromosome 10 (LOC_Os10g10580), chromosome 11 (LOC_Os11g38020) and chromosome 12 (LOC_Os12g03160, LOC_Os12g17750) (Table 2, Figure 3B). These QTNs were identified as major QTNs with higher genetic heritability, which are validated by two or more independent methods namely, FASTmrEMMA, ISIS_EM-BLASSO, mrMLM, and pLARmEB (Table 2, Figure 3B). Of the 9 QTNs associated with hardness, none of the QTNs was identified to affect both hardness and AC. A prominent QTN snp_12_10170782 (C>T) was identified in the promoter region of LOC_Os12g17750 (unknown function) identified for influencing hardness using three independent methods pLARmEB, ISIS_EM-BLASSO, mrMLM with LOD score of 6.3, 8.7, and 10.24 (Table 2 and Table S2). For the candidate gene LOC_Os12g17750, reference haplotype CTAC being abundant in major *Indica* germplasm with intermediate hardness and its alternative haplotype TCCG representing lines depicted higher hardness value (Figure 3C). Likewise, additional QTNs representing missense mutations such as snp_05_12361761 leading to amino acid change (Val>Ile) in candidate LOC_Os05g21010 and snp_12_1204320 (Gly>Asp) was detected in LOC_Os12g03160, associated with HRD trait (Table 2).

Genetic basis of springiness textural trait was defined through SL-GWAS on chromosome 2 (LOC_Os02g39630) validated using ML-GWAS approach (Table 1, Figure 4). Additional QTN identified through SL-GWAS resulted in identifying a locus LOC_Os09g34340 with contrasting haplotypes (CGCA with higher value of SPR and CACG with lower value of SPR). Employing ML-GWAS approach 5 additional QTNs were identified and among them LOC_Os12g22020 locus was defined by contrasting haplotype TCCAGGAGG with higher SPR value and alternative haplotype TCCGAGGGG containing lines possess lower SPR (Figure 4). We also identified snp_01_3464018 causing premature termination at start codon of the candidate locus LOC_Os01g07330 (unclassified), which exhibited the distinct haplotypes showing variation for the SPR (Figure 4C). Moreover, the extreme haplotypes detected for ADH, HRD, and SPR also showed the consistent phenotype across wet and dry seasons (Figure S4).

For COH, we observed significant snp_10_13693521 located downstream of LOC_Os10g26370 validated through ISIS_EM-BLASSO, FASTmrEMMA multi-locus GWAS methods with LOD score value of 3.7 and 5.7 (Table 2). Additional QTN snp_12_12385384 identified from ISIS_EM-BLASSO was found to associate very significantly with COH (LOD 6.35 with *r*^2^ 12.05) and SPR (LOD 4.95 with *r*^2^ 2.88).

Kyota Encyclopedia of Genes and Genomes (KEGG) analysis was conducted to identify the functional categories across all the QTNs identified. A total of 40 candidate genes with functional information were mined, of which 17 were involved in genetic information processing, whereas the rest of the candidate genes were involved in other cellular and metabolic processes (Figure S6).

## Discussion

### Interlinking amylose content variation with textural attributes

Texture of cooked rice plays a pivotal role in consumer acceptability; henceforth researchers continuously develop strategies to predict texture of cooked rice. AC is widely explored to capture the diversity of rice quality (Anacleto et al., 2015) in rice breeding programs. The challenge lies when rice varieties within similar AC quality class are easily differentiated by consumers (Champagne et al., 1999, 2010), and thus secondary traits derived based on AC versus GT or AC versus GC will be prioritized. This situation highlights the importance of secondary assays that could further differentiate rice varieties into distinct quality classes. It is assumed that rice varieties within the same AC and GC ranges are distinguished from each other for textural attributes. Mega varieties which have been developed during 1965–1990 were found to possess unique textural attributes, which cannot be distinguished alone by AC. Hence revealing textural attributes is a crucial step in fine-tuning product profiles for capturing major rice markets tend to distinguish rice within intermediate and high AC. Multi-modal descriptive sensory description is the ultimate reference to distinguish textural features of rice varieties (Anacleto et al., 2015). However, it is difficult to routinely implement descriptive sensory methods to capture textural preferences for selecting breeding material from rice improvement programs due to large number of samples, low throughput methodology. Thus to develop and deliver selection tools to breeders, there is a need to bridge proxy grain quality selection tools (AC) with instrumental based TPA analysis and predict textural ideotypes from diverse germplasm (Champagne et al., 1999).

TPA is an semi-throughput approach used to measure the mechanical response during a double compression, which mimics first and second bite of a food sample (Stokes et al., 2013). Until now, attempts have been made to correlate instrumental texture profiles with various rice quality predictors (Ohtsubo et al., 1990; Champagne et al., 1999, 2004). Among textural attributes, HRD is considered as an important attribute of cooked rice texture strongly affect the consumer acceptance (Perez et al., 1979; Li et al., 2016). We employed highly diverse *Indica* germplasm enriched with intermediate to high AC to generate phenotyping data of various textural attributes using TPA (Figure 1). These results suggest a week positive correlation between AC and HRD (Figure 1). This is differed with the outcome of previous studies where HRD showed very significant positive correlation with AC (Perez et al., 1979; Bao et al., 2004; Li et al., 2016, 2017). These results have proved inconsistent and at times coincidental, most likely because of utilization of small sample sets to establish associations. In addition, the samples are most likely not representative of covering the entire breadth and depth of the diversity of *Indica* germplasm.

Our results delineated that AC was negatively correlated with the ADH (*r* = −0.83). Previous studies reported that low AC value with higher amylopectin content increase the stickiness in cooked rice (Juliano, 1992; Reddy et al., 1993; Windham et al., 1997; Li et al., 2016). Additionally, AC displayed non-significant correlation with COH, SPR, which is in agreement with the Cho et al. (2010). These results suggested that AC is not the sole determinant of cooked rice texture, and it is important to equally consider other component textural attributes viz. HRD, COH, and SPR. Moderate to high correlations exist among HRD, COH, and SPR traits, which suggest the existence of interdependence among these three textural traits. To support future breeding programs, we need to dissect genetics and leverage the gene discovery attempts toward crop improvement of textural attributes.

### Dissecting the genetic basis of texture related traits

Genetic dissection of the textural attributes has been explored in previous studies using *Japonica*/*Indica* biparental mapping population (Bao et al., 2002, 2004; Cho et al., 2010). Nevertheless, studies involving high resolution dissection of genetic basis of important texture related traits are very limited. To estimate the heritability, we have measured amylose content from two independent years and used this phenotyping data to identify the genetic regions. We identified the same genomic regions on chromosome 6, regulating amylose content across two season data with higher heritability's values (*h*^2^ = 0.85 and 0.86), reflecting the consistency across both seasons (Figure S3). As the selected diversity panel showed high phenotypic variation for amylose, which influences texture, we have used the data for textural parameters from 2014 dry season (in randomized complete block design) for conducting the GWAS. The texture based phenotyping data were generated from nine technical replicates. In this approach we have considered the ample replications to ensure the phenotype consistency and explored the diversity population covering vast range of phenotypic variation for textural attributes to define its genetic basis. In present study, AC and ADH have reflected the high narrow sense heritability (*h*^2^ = 0.86). Thus the defined genetic region will be an added value. Furthermore, notably AC and ADH showed high degree of correlation and overlapping genetic region influenced by both traits. Likewise, HRD (*h*^2^ = 0.82) and COH (*h*^2^ = 0.68), showed higher heritable values from the RDP than that observed in previous studies dealing with biparental mapping populations (Bao et al., 2002; Cho et al., 2010). In addition, we selected the accessions possessing the haplotypes exhibiting the extreme phenotypes for adhesiveness, hardness and springiness, and phenotyped for different textural attributes in the seed lots collected from two different seasons. As a result, we identified the values from both of the seasons close and comparable to each other (Figure S4). These results further confirmed that genetic component majorly regulating the textural trait is highly heritable and less affected by the environmental effects. In addition, we have utilized days to maturing data as covariate and re-run single locus GWAS for AC and four textural attributes. All the genetic regions identified for textural attributes using SL-GWAS peaks for AC, ADH, HRD, and SPR remains significant, when we run with days to maturity as covariate (refer Figure S5). Through this approach we rule out any influence of days to maturity on texture in the currently studied core collection panel.

EMMA algorithm has been extensively used to dissect the complex traits due to its robustness and reliability. Furthermore EMMA model corrects for confounding effects of subpopulation structure and relatedness between individuals (Kang et al., 2008; Kumar et al., 2015; Campbell et al., 2017). Application of single-locus scan approach under polygenic background with diverse population structure controls do not facilitate the detection of small effect QTNs, as the model fails to consider the integrated effect of multiple markers under specific loci (Zhang et al., 2018). Alternative and more powerful approaches for marker-trait association have been developed to address the shortcomings of one dimensional scan. Hence in the present study we used four multi-locus model approaches FASTmrEMMA (Wen et al., 2018), pLARmEB (polygenic-background-control-based least angle regression plus empirical Bayes) (Zhang et al., 2017), mrMLM (Wang et al., 2016), and ISIS_EM-BLASSO (Tamba et al., 2017) to conduct GWAS analysis in order to capture minor QTNs related to texture traits. A total of 224 SNPs associated with AC and textural attributes such as ADH, COH, HDR, and SPR were defined using ML-GWAS method. When comparing the four multi-locus methods, a high number of 97 SNPs were validated with at least 2 out of the four multi-locus methods. Among them 48 novel loci were defined to influence texture attributes. Among the implemented ML-GWAS methods, pLARmEB and ISIS EM-BLASSO detected the higher number of significant QTNs. The same observation was made in the recent study (Sant'ana et al., 2018), who reported a high number of trait-associated SNPs using two methods. On the other hand (Ma et al., 2018), acknowledge the robustness of ISIS EM-BLASSO than the other three methods (FASTmrEMMA, pLARmEB, mrMLM). More than half of the QTN detected were specific to one of the four methods used. Thus validation accounted by combinatory approaches of ML-GWAS methods has been considered in our study for interpreting biological inferences of rice texture.

Using the efficient mixed-model association we identified 10 robust QTNs with major effect QTNs significantly associated with texture-related traits validated using SL-GWAS and ML-GWAS approaches. To discover medium and small effect QTNs, we used confirmatory validation through at least two independent methods of ML-GWAS approaches. Unlike SL-associations, ML-association approaches are considered as effective in taking the joint effects of multiple genetic markers into account and avoid any stringent criterion leading to likelihood of missing out functionally relevant genomic loci (Wang et al., 2016; Tamba et al., 2017; Wen et al., 2018). Indeed, although, no SNP significantly associated QTNs were identified for cohesiveness using SL-GWAS and mrMLM methods, we detected putative genomic regions underlying COH using three other ML-GWAS methods. Multi-locus GWAS has gained popularity with a growing number of studies reporting the use of this approach (Misra et al., 2017; Ma et al., 2018; Sant'ana et al., 2018; Zhang et al., 2018) to perform marker-trait association. Some of the distinct advantages of multi-locus GWAS over single-locus GWAS are their power, accuracy in QTN effect estimation, reduced rate of false positives (Wang et al., 2016; Tamba et al., 2017; Ma et al., 2018).

We identified a well characterized major effect QTN affecting AC and ADH within *GBSS1* region confirmed through SL- and ML-GWAS, particularly loci at the 5′-splice site of first intron (Figure 2), which is in agreement with previous reports (Hsu et al., 2014; Yang et al., 2014; Wang et al., 2017). Furthermore, two additional SNPs were detected for regulating AC, earlier identified by Butardo et al. (2017) for determining AC. In addition to *GBSS1, w*ithin the hot spot QTL of chromosome 6 linking AC with ADH textural attribute we identified additional loci influencing texture traits. For instance, haplotypes derived from candidate genes encoding alpha/beta-hydrolases, which belongs to largest group of structurally related enzymes (Holmquist, 2000) and uncharacterized phosphotransferase, explicitly showed the phenotypic variation with AC and ADH traits. Notably, adjacent region of *GBSSI* reflected the variations for ADH has been reported previously but the candidate loci were not unraveled (Wang et al., 1995; Isshiki et al., 1998). This may be attributed to low resolution owing to limited recombination events observed in case of bi-parental population.

Besides *GBSSI*, we did not detect the QTNs from candidates involved in starch biosynthesis, namely starch synthase IIa (*SSIIa*) (Umemoto et al., 2002; Nakamura et al., 2005), starch branching enzyme (*SBE IIb*) (Nishi et al., 2001; Tanaka et al., 2004; Nakata et al., 2018), as the RDP panel employed in the present study belongs to *indica* population. From the past studies, variation in *SSIIa* alleles were identified by exploring inter species genetic variation between *indica* compared to *japonica*, due to enrichment of the intermediate amylopectin chains (DP 12–24) (Umemoto et al., 2002, 2004). Similarly, SBEIIb revealed to possess different alleles in two subgroups *indica* and *japonica* (Luo et al., 2015). Allelic variants of both of the genes can markedly distinguish respective favored allele in *indica* vs. *japonica* germplasm. In the present study, underlying large effect candidate genes influencing ADH alone, but not AC, related to the identification of candidate gene metallic protease involved in the protein degradation and the allergenic protein, which might potentially involve in regulation of the structural proteins determining texture of cooked rice. In previous studies, protein content was correlated negatively with the adhesiveness (Lyon et al., 2000) and other rice texture attributes (Champagne et al., 1999; Martin and Fitzgerald, 2002). Besides the textural traits being highly correlated among each other, we also detected common QTNs between AC and ADH (Tables 1, 2). Additionally, several significant SNPs involved in textural attributes found to influence alternative splicing were identified in this study, which suggests the importance of post-transcriptional regulation. Since most of the genes involved in the core regulatory pathways, the functional characterization of novel candidate genes with non-synonymous amino acid alteration influencing various textural attributes of ADH, HRD, and COH traits will be worth exploring its functional validation through transgenic studies. These novel haplotypes defined in the present study will serve as important genetic resource for future breeding strategies to capture textural attributes.

In summary, our findings addressed the underlying genetic basis of rice texture attributes. We found considerable phenotypic variations in texture attributes (adhesiveness, hardness, springiness, cohesiveness, and amylose content) among the 218 *indica* accessions. Highly negative correlation between amylose content and adhesiveness was observed, which could explain the fact that rice with low amylose is stickier. We identified multiple major/minor QTNs linked with rice cooking properties using SL-GWAS, followed by ML-GWAS using 4 independent methods (FASTmrEMMA, ISIS_EM-BLASSO, mrMLM, and pLARmEB). An important hot spot QTLs on chromosome 6 where QTNs for ADH co-localized with QTNs for AC were identified. Furthermore, a fine mapped genetic region on chromosome 8 affecting HRD was identified. The use of different models increased the number of variations captured across the diverse germplasm lines. Multi-locus model using different methods could overcome the limitations of single-locus analysis. Furthermore, this integrative approach has enabled the identification of novel small and large effect putative potential candidate genes and diagnostic haplotypes, which subsequently on validation, potentially be deployed in breeding to improve rice texture.

## Author contributions

NS conceptualized the research work, designed and supervised all experiments, wrote and edited the manuscript. GM conducted GWAS and haplotype analyses, generated figures, and created an in-house functional annotation pipeline. SB and EM interpreted the data, prepared excel sheets, and developed the first draft of the manuscript. CD and RC conducted texture profile experiments, developed TPA methodology and processed the raw data. CL conducted texture profile experiments from different seasons for the contrasting haplotype lines.

### Conflict of interest statement

The authors declare that the research was conducted in the absence of any commercial or financial relationships that could be construed as a potential conflict of interest.
