# Supplementary material for: Deciphering the Genetic Architecture of Cooked Rice Texture
Source: Front Plant Sci. 2018 Oct 2;9:1405. doi: 10.3389/fpls.2018.01405 (PMC6176215; doi:10.3389/fpls.2018.01405)
Supplement: Supplementary file 2 [file Presentation_1.pdf]

Deciphering the Genetic Architecture of Cooked Rice Texture

Gopal Misra, Saurabh Badoni, Cyril John Domingo, Rosa Paula O. Cuevas, Cindy Llorente, Edwige Gaby Nkouaya Mbanjo and Nese Sreenivasulu

International Rice Research Institute, Metro Manila, Philippines

Supplementary Figures

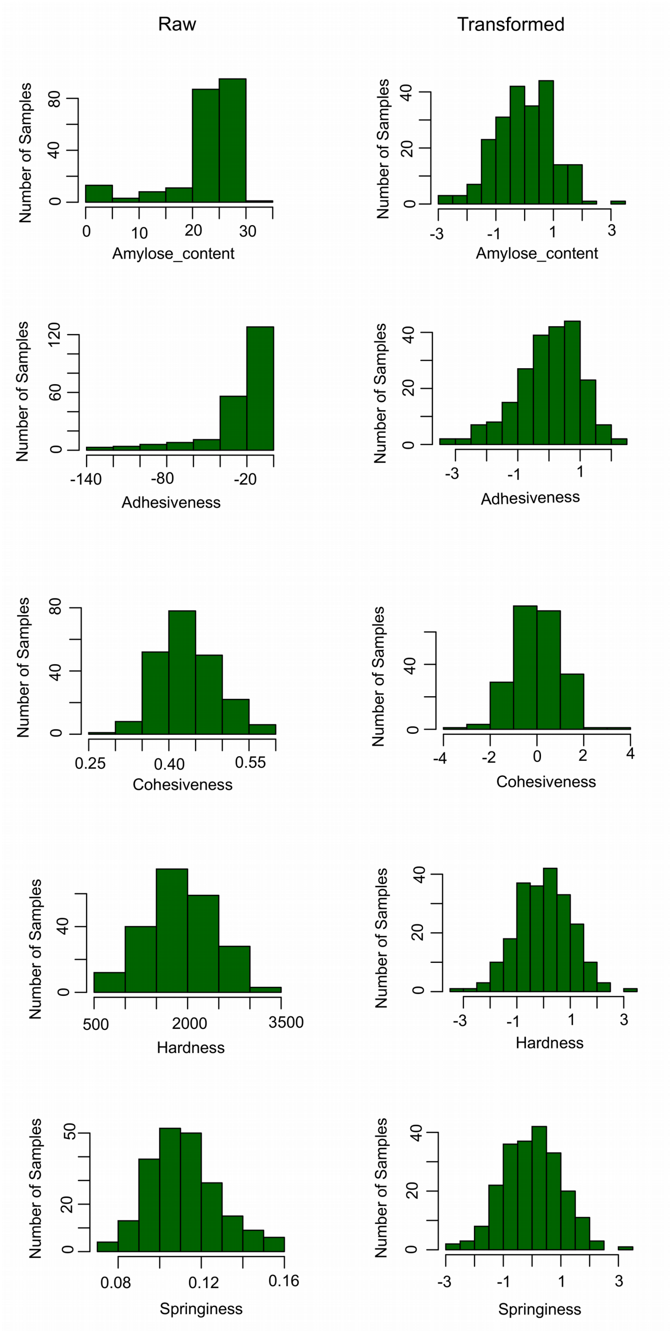

Figure S1: Raw and transformed phenotype distribution plotted as histogram. Left column represented raw phenotype distribution while right column showed as transformed phenotype distribution.

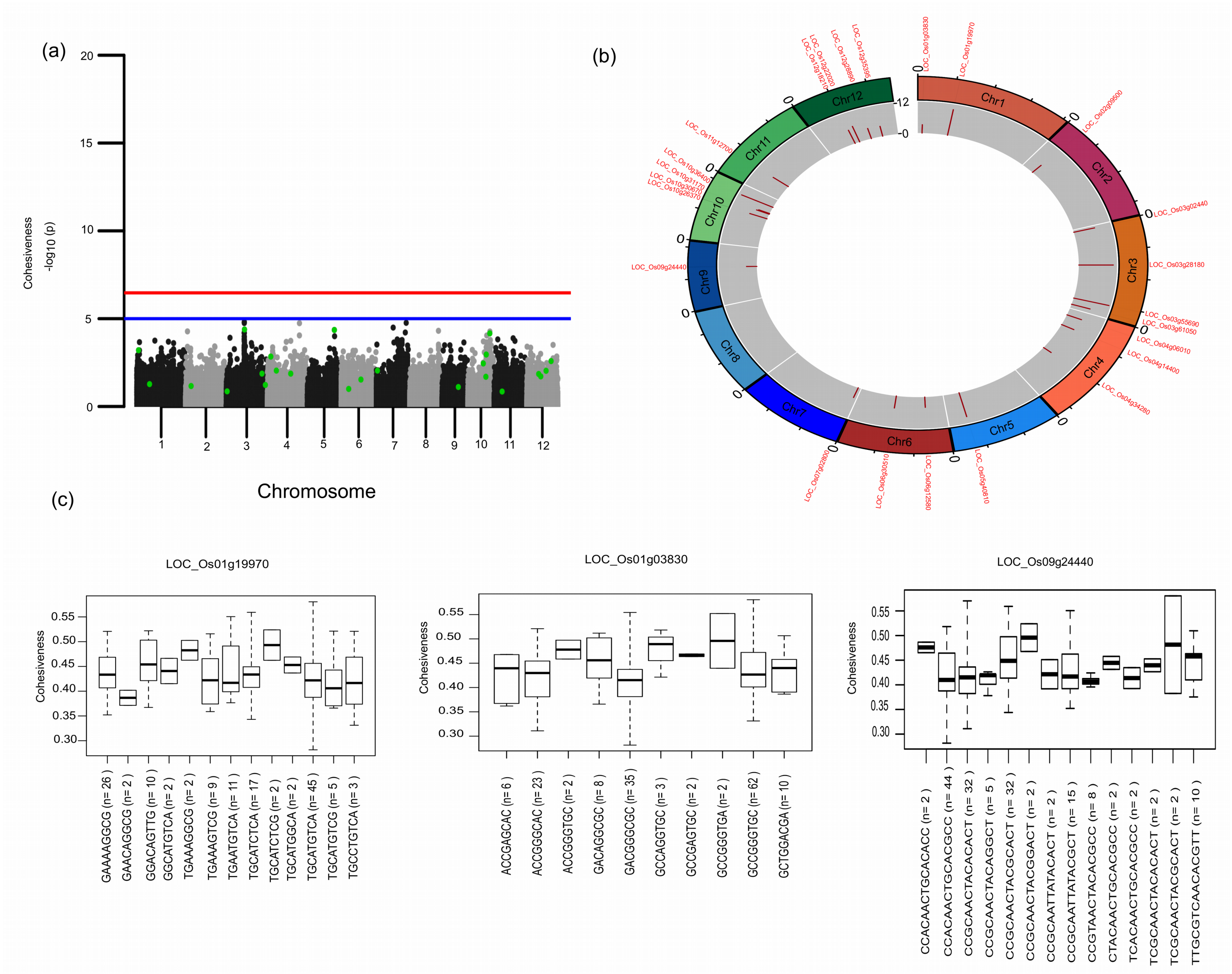

Figure S2: Genetic regions identified through ML-GWAS and SL-GWAS for cohesiveness (COH). **(a)** Single locus association overlaid with multi-locus associations (QTNs) shown as green dots with significant threshold of LOD ≥ 3 score. **(b)** Circos representing the physical positioning of 12 chromosomes with locus IDs of significant QTNs identified in ML-GWAS, followed by depiction of LOD score in the innermost circle. **(c)** Phenotypic distribution of haplotypes shown as boxplot for selected genes identified from ML-GWAS method.

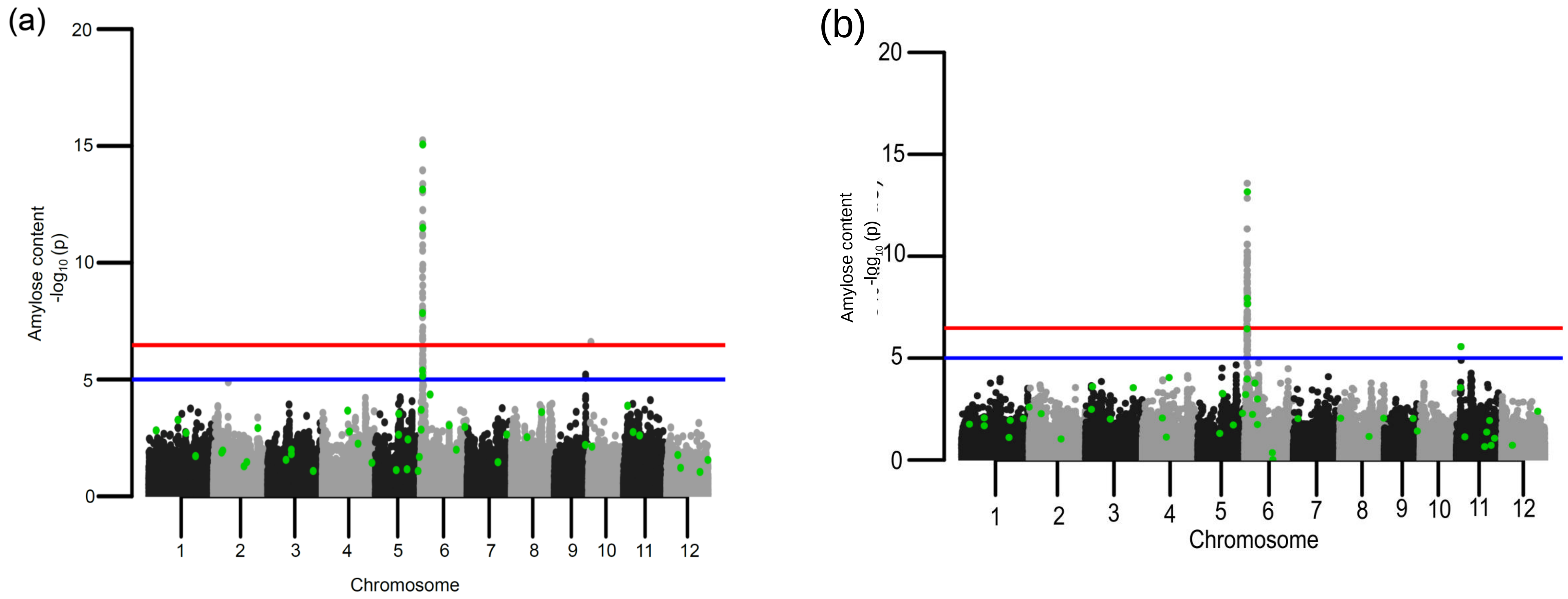

Figure S3 : GWAS conducted for AC under two different seasons 2014ds **(a)** and 2014ws **(b)**. Genome-wide significant threshold line [$-\log_{10}(P)$ = 6.47] is drawn as red, whereas suggestive line is represented by blue line at $-\log_{10}(P)$ of 5. The heritability values were observed consistent across both of the seasons (h^2 = 0.86 in 2014ds, and h^2 = 0.85 in 2014ws).

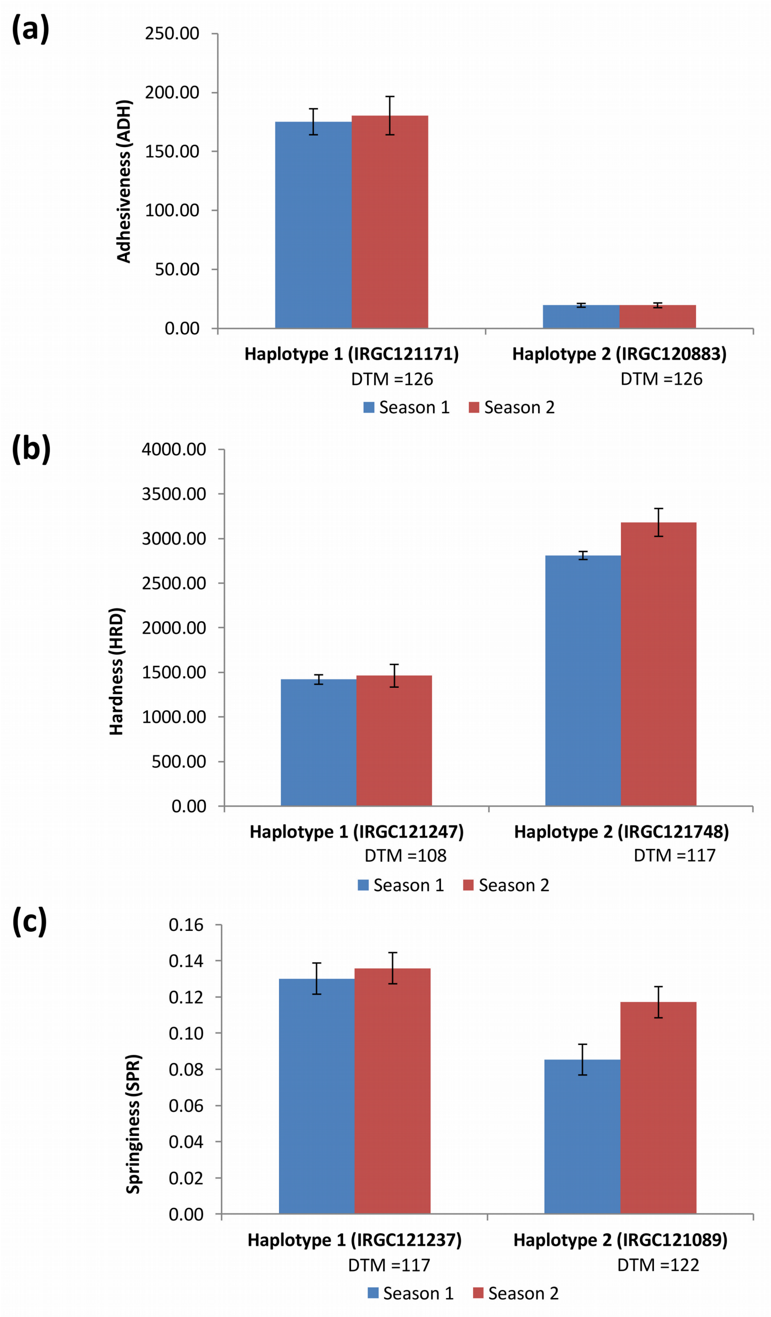

Figure S4: The behavior of key textural traits in two different seasons generated from lines with contrasting haplotype exhibiting the respective extreme phenotypic value. **(a)** Adhesiveness (ADH) trait was assessed in season 1 (2014 dry season) and season 2 (2015 dry season/2014 wet season) in lines harboring haplotype 1 (CCT) and haplotype 2 (CTT). Likewise, **(b)** Hardness (HRD) was measured between two independent seasons from contrasting lines possessing the haplotype 1 (CTAC) and haplotype 2 (CTAC), whereas **(c)** springiness was identified in lines with haplotype 1 (TCCAGGAGG) and haplotype 2 (TCCGAGGGG). Standard error is represented as the error bars.

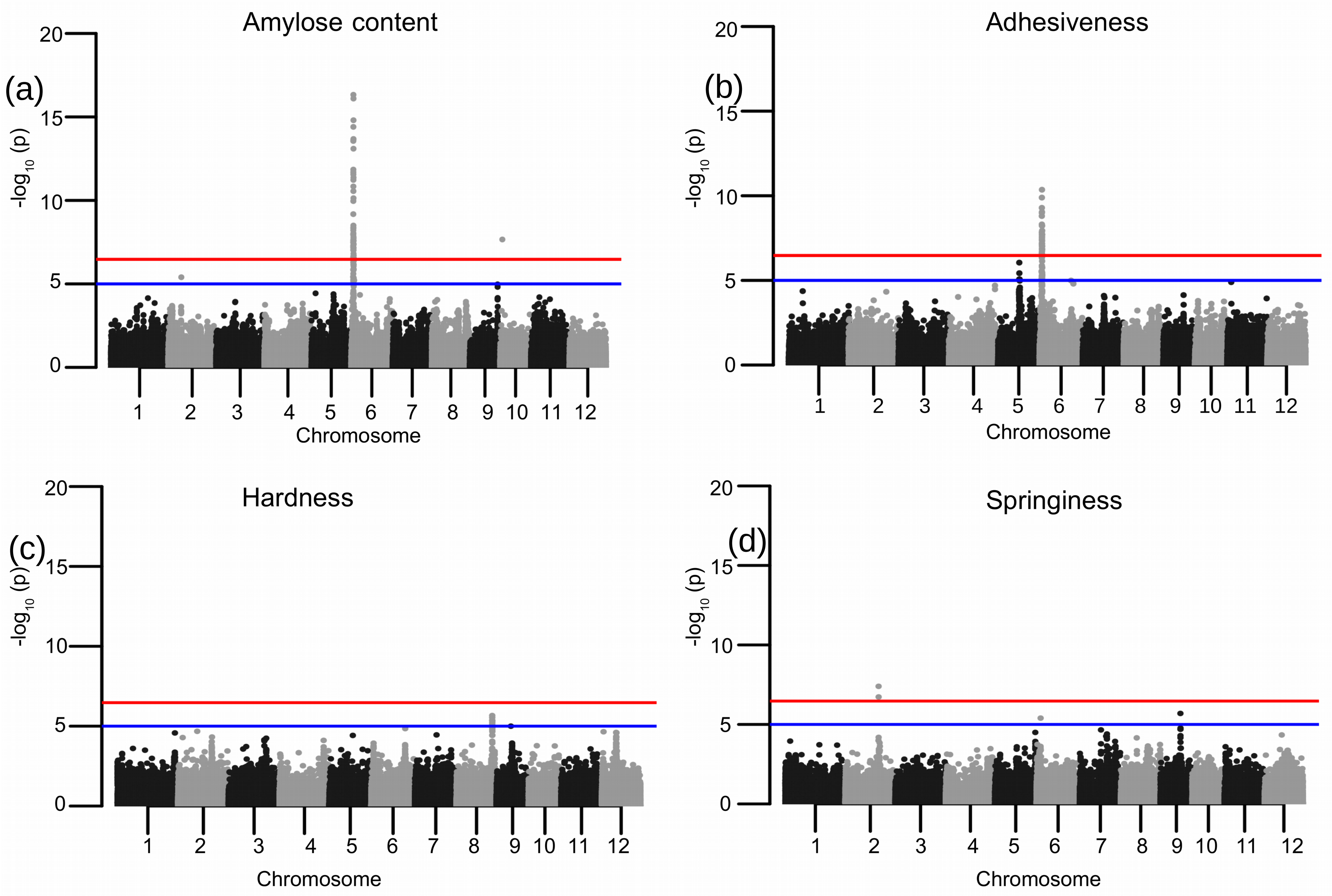

Figure S5: GWAS conducted for textural traits including AC, ADH, HDR, and SPR, while running days to maturity (DTM) as covariate. All of the prominent peaks for different textural traits-including amylose content **(a)**, adhesiveness **(b)**, hardness **(c)** and springiness **(d)**, were mapped in similar genomic regions as identified through earlier GWAS ran without covariate. This further suggests no influence of DTM on texture in the current core collection panel. Genome-wide significant threshold line [–log 10 (P) = 6.47] is drawn as red, whereas suggestive line is represented by blue line at –log 10 (P) of 5.

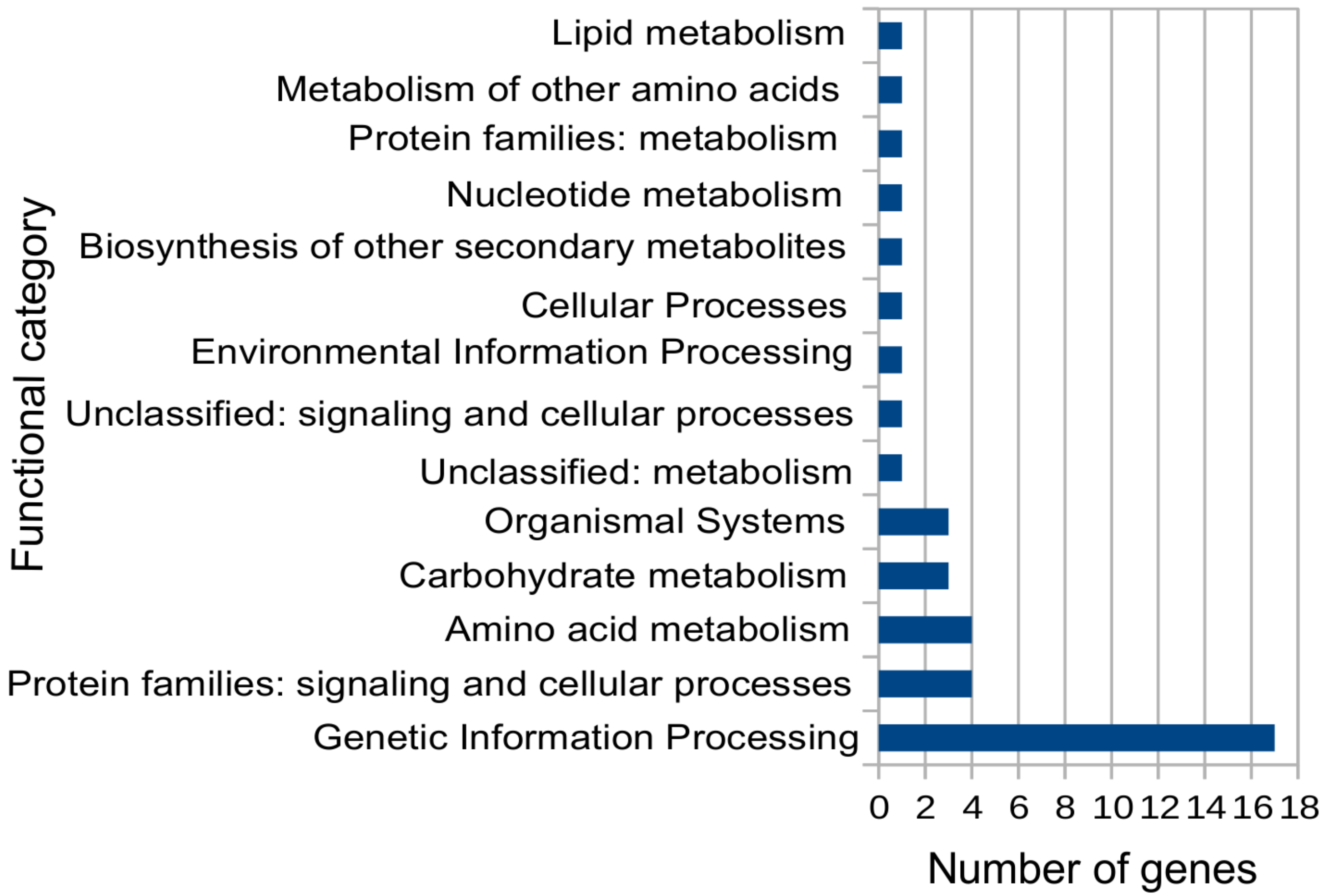

Figure S6 : KEGG analysis for the candidate genes identified in multi-locus GWAS for textural attributes and AC. A total of 40 candidate genes identified as significant QTNs were classified for the functional categories using the KEGG pathway chart.

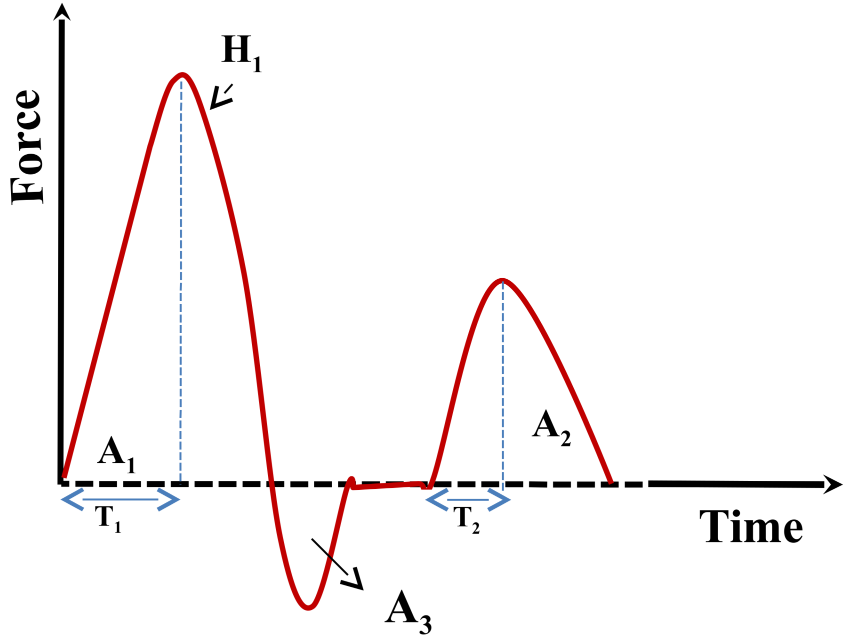

Adhesiveness (ADH) = Negative force area under the first bite (A3).

Hardness (HRD) = Maximum force required (showed as H_1) for first compression cycle, simulating the force required to press the food using molars. Signifies the peak force of the first compression by the height of first curve.

Cohesiveness (COH) = Ratio of the positive force areas under second compression to that of first one (A_2/A_1).

Springiness (SPR) = Springiness (represented as T_2/T_1) is the height that the food recovers during the time that elapses from the upstroke to the peak in the second curve (T_2) to the time elapsed from starting point to the peak of the first curve (T_1), representing degree of sample height recovery.after partial compression.

Supplementary Note: Representation and definition of different textural attributes as detected in TPA (Texture profile analyzer)
